# Inter-rater variability for the American Society of Anesthesiologists classification in patients undergoing hepato-pancreato-biliary surgery (MILESTONE-2): international survey among surgeons and anaesthesiologists

**DOI:** 10.1093/bjsopen/zrae162

**Published:** 2025-02-28

**Authors:** Simone Augustinus, Jasper P Sijberden, Matthanja Bieze, Vandana Agarwal, Luca A Aldrighetti, Adnan Alseidi, Francisco C Bonofiglio, Kevin C P Conlon, Katia Donadello, Joris Erdmann, Cristina Ferrone, Michael Guertin, Ronald Harter, Maria E Franceschetti, Guiseppe K Fusai, Bas Groot Koerkamp, Thilo Hackert, Jin-Young Jang, Thomas Kander, Tobias Keck, Dominik Krzanicki, Ho-Jin Lee, Keith Lewis, Giuseppe Natalini, Carla Nau, Timothy M Pawlik, Henry A Pitt, Rafaella Reineke, Roberto Salvia, Eduardo de Santibanes, Shailesh V Shrikhande, Martin Smith, Attila Szijarto, Bobby Tingstedt, Alice C Wei, John Windsor, Mohammed Abu Hilal, Manuel Pardo, Markus W Hollmann, Marc G Besselink

**Affiliations:** Department of Surgery, Amsterdam UMC, Location University of Amsterdam, Amsterdam, The Netherlands; Cancer Center Amsterdam, Amsterdam, The Netherlands; Department of Surgery, Amsterdam UMC, Location University of Amsterdam, Amsterdam, The Netherlands; Cancer Center Amsterdam, Amsterdam, The Netherlands; Department of Surgery, Fondazione Poliambulanza Istituto Ospedaliero, Brescia, Italy; Department of Anesthesiology, Amsterdam UMC, Location University of Amsterdam, Amsterdam, The Netherlands; Department of Anesthesiology and Pain Management, Toronto General Hospital, University of Toronto, Toronto, Ontario, Canada; Department of Anesthesia, Critical Care and Pain, Tata Memorial Hospital, Homi Bhabha National Institute, Mumbai, Maharashtra, India; Hepatobiliary Surgery Division, Ospedale San Raffaele, Milano, Italy; Division of Hepatopancreatobiliary and Endocrine Surgery, University of California, San Francisco, California, USA; Department of Anesthesiology, Hospital Italiano de Buenos Aires, Buenos Aires, Argentina; Department of Surgery, Trinity College Dublin, Tallaght University Hospital, Dublin, Ireland; Department of Anesthesia and Intensive Care B, DSCOMI, University of Verona, University Hospital Integrated Trust of Verona, Verona, Italy; Department of Surgery, Amsterdam UMC, Location University of Amsterdam, Amsterdam, The Netherlands; Cancer Center Amsterdam, Amsterdam, The Netherlands; Department of Surgery, Massachusetts General Hospital, Harvard Medical School, Boston, Massachusetts, USA; Department of Anesthesiology, The Ohio State University Wexner Medical Center & College of Medicine, Columbus, Ohio, USA; Department of Anesthesiology, The Ohio State University Wexner Medical Center & College of Medicine, Columbus, Ohio, USA; Department of Anesthesia and Intensive Care, Istituto Fondazione Poliambulanza, Brescia, Italy; Hepatobiliary Surgery and Liver Transplantation Unit, Royal Free Hospital, London, UK; Department of Surgery, Erasmus MC, University Medical Center Rotterdam, Rotterdam, The Netherlands; Department of General, Visceral and Thoracic Surgery, University Hospital Hamburg-Eppendorf, Germany; Department of Surgery and Cancer Research Institute, Seoul National University College of Medicine, Seoul, South Korea; Department of Intensive and Perioperative Care, Skåne University Hospital, Lund and Lund University, Lund, Sweden; DGAV StuDoQ|Pancreas and Clinic of Surgery, UKSH Campus Lübeck, Lübeck, Germany; Department of Anaesthesia, Royal Free Hospital, London, UK; Department of Anesthesiology and Pain Medicine, Seoul National University Hospital, Seoul, South Korea; Department of Anesthesiology and Perioperative Medicine, Rutgers Robert Wood Johnson Medical School, New Brunswick, New Jersey, USA; Department of Anesthesia and Intensive Care, Istituto Fondazione Poliambulanza, Brescia, Italy; Department of Anaesthesiology and Intensive Care, University Medical Centre Schleswig-Holstein, Lübeck, Germany; Department of Surgery, The Ohio State University Wexner Medical Center, Columbus, Ohio, USA; Department of Surgery, Rutgers Cancer Institute of New Jersey, New Brunswick, New Jersey, USA; Department of Anesthesiology and Intensive Care, IRCCS San Raffaele Scientific Institute, Milan, Italy; General and Pancreatic Surgery Department, Pancreas Institute, University and Hospital Trust of Verona, Verona, Italy; Department of Surgery, Hospital Italiano, University of Buenos Aires, Buenos Aires, Argentina; Department of GI and HPB Surgery, Tata Memorial Centre, Homi Bhabha National Institute, Mumbai, India; Department of Surgery, Faculty of Health Sciences, School of Clinical Medicine, University of the Witwatersrand, Johannesburg, South Africa; Department of Anesthesiology, I. sz. Sebészeti Klinika, Semmelweis Egyetem, Budapest, Hungary; Department of Surgery, Clinical Sciences Lund, Lund University, Skåne University Hospital, Lund, Sweden; Hepatopancreatobiliary Service, Department of Surgery, Memorial Sloan Kettering Cancer Center, New York, New York, USA; HBP/Upper GI Unit, Auckland City Hospital/Department of Surgery, University of Auckland, New Zealand; Department of Surgery, Fondazione Poliambulanza Istituto Ospedaliero, Brescia, Italy; Department of Anesthesia and Perioperative Care, University of California, San Francisco, California, USA; Department of Anesthesiology, Amsterdam UMC, Location University of Amsterdam, Amsterdam, The Netherlands; Department of Surgery, Amsterdam UMC, Location University of Amsterdam, Amsterdam, The Netherlands; Cancer Center Amsterdam, Amsterdam, The Netherlands

## Abstract

**Background:**

Patients undergoing hepato-pancreato-biliary surgery are typically preoperatively assessed using the American Society of Anesthesiologists (ASA) classification, which is also used for case-mix adjustment when comparing centre outcomes. Studies determining the inter-rater variability of the ASA classification within hepato-pancreato-biliary surgery are currently lacking.

**Methods:**

An international survey was collected and a case-vignette study was performed (November 2022–April 2023) regarding the ASA classification in patients undergoing hepato-pancreato-biliary surgery among anaesthesiologists and surgeons from (inter)national societies. The survey consisted of 23 questions and eight case-vignettes. Primary analysis included descriptive statistics and the inter-rater variability was calculated using Light's Kappa.

**Results:**

Overall, 1283 participants from 55 countries responded: 1073 (84%) anaesthesiologists and 210 (16%) surgeons. The ASA classification was commonly used, both clinically 1003/1283 (78%) and for research 728/762 (96%). The majority of respondents (*n* = 1019, 79%) declared that ASA score impacted their perioperative strategy. There inter-rater variability was fair–moderate (Kappa 0.26–0.42) in all case-vignettes. Inter-rater variability differed within and among geographic regions for each case. Over 80% (*n* = 1138) of respondents stated that they would take the underlying disease (for example cancer) into account, but this changed the preferred ASA score within the case-vignettes by only 1%. Type of surgery changed the preferred score in the case-vignettes (13% difference). The most common suggestions to improve the ASA classification were to clarify whether type of operation should be considered, create a more extensive definition, and provide more examples.

**Conclusions:**

Inter-rater variability was present within the ASA classification of patients undergoing hepato-pancreato-biliary surgery, which may impact perioperative strategy and hamper research results. Additional guidance to classify patients according to ASA is urgently needed. Until then, more objective measurements should be considered for case-mix adjustment within research.

## Teaser

This is an international survey and case-vignette study including 1283 participants from 55 countries. Interrater variability regarding the ASA classification in patients undergoing HPB surgery was observed, which may impact perioperative strategy. Additional guidance to classify patients according to ASA is urgently needed. Until then, more objective measurements should be considered for case-mix adjustment within research.

## Introduction

For over 60 years, the American Society of Anesthesiologists (ASA) physical status classification system has been used to facilitate bedside assessment and categorize patients’ co-morbidities prior to anaesthesia^1^. Combined with other factors (for example type of surgery, frailty, level of deconditioning) the classification can be used to predict perioperative risks. Nevertheless, considerable variability in ASA scoring among anaesthesiologists has been recognized. Several case-vignette studies addressing common surgical procedures in individual countries have demonstrated significant inter-rater variability^2–5^.

Hepato-pancreato-biliary (HPB) surgery is one of the most complex types of abdominal surgery and is associated with considerable perioperative risks^6,7^. A recent study identified a large variability in ASA classification among four large registries for pancreatic surgery; classification as ASA III/IV varied from 78% (North America), 48% (Germany), 23% (the Netherlands), to 3% (Sweden) of patients^8^. As large differences in patients’ co-morbidities among these four countries are highly unlikely, this large discrepancy could partly arise from a different interpretation of the ASA classification between countries. For example, whether the type of surgery (for example more or less complex) or underlying disease (for example malignancy) is accounted for when determining the ASA score. Alternatively, other variables might explain the different ratings in ASA classification (for example reimbursement policies, experience within a centre). It is of importance to understand the reasons behind these differences as ASA score is increasingly used for case-mix adjustment when comparing surgical outcomes among centres in clinical auditing^9–12^.

Data on the reliability and inter-rater variability of the ASA classification among patients undergoing HPB surgery are lacking. The first MILESTONE survey, conducted among surgeons and anaesthesiologists, assessed worldwide variety in fluid and pain management in liver surgery^13^. This second MILESTONE survey among anaesthesiologists and surgeons aims to define international inter-rater variability, as well as characterize potential explanations for any observed variability among patients undergoing HPB surgery. Such data will hopefully help to optimize the ASA classification and provide context for ASA-related benchmarking of postoperative outcomes.

## Methods

### Study design

An online survey including clinical case-vignettes was created by the multidisciplinary MILESTONE study group and followed the AAPOR best practices for survey research^14^. The Medical Ethical Committee of the Amsterdam UMC confirmed that the Medical Research Involving Human Subjects Act (WMO) did not apply to the study. Participants were advised that they provided informed consent to the use of their anonymized responses by completing and submitting the survey.

### Population

Multiple national and international societies were invited via email to participate and disseminate the survey among their members. For anaesthesiologists, 133 national anaesthesiology societies associated with the World Federation of Societies of Anaesthesiologists (WFSA) and the Liver Intensive Care Group of Europe (LICAGE) were invited. For surgeons, the International Hepato-Pancreato-Biliary Association (IHPBA) and its three regional chapters, four minimally invasive HPB surgery societies/groups, and three regional HPB societies were asked to participate. A full list of invited societies can be found in *Supplementary Material S1*. The co-authors were asked to distribute the survey online in their professional networks.

### Survey distribution

The survey was created by the MILESTONE study group (that is all authors), a multidisciplinary group of experts on perioperative care for HPB patients. The survey was tested by the first and senior authors by completing the survey and checking for completeness and potential flaws before sending it to the societies. When societies wished to collaborate, the survey link was disseminated by email to the members between November 2022 and April 2023. Data were collected anonymously using Qualtrics XM survey software® (Qualtrics, Provo, Utah, USA).

### Data collection

The survey gathered demographic information on country, sex, age, medical specialty, years of experience, and level of expertise present in the hospital. Subsequent questions included six general questions on the ASA classification, nine questions on considerations made in the ASA classification process, and eight clinical cases (full survey: *Supplementary Material S2*). Within the clinical cases, four patients undergoing pancreatoduodenectomy (PD) and four patients undergoing extended left hemihepatectomy without biliary reconstruction were described. In each case a single aspect changed, which was highlighted in bold (*Supplementary Material S2*). The respondents were asked to score the ASA classification for each case (that is ASA I–V). Within the MILESTONE study group consisting of international experts, the aspects that were tested in the case-vignettes were based on well-known points of discussion from clinical practice. Some of these were addressed both within the case-vignettes and within the general questions, to confirm whether the respondents’ opinion on how the classification should be interpreted are in line with how they classify the patients within the case-vignettes. An open question was added to determine if some discussion points were missed. Points of discussion that could not be incorporated within a clinical case were incorporated in the general questionnaire (that is financial compensation, experience in a peripheral/academic centre). No ‘correct ASA scores’ per clinical case were described, as there is no consensus yet within the ASA classification on the points up for discussion.

### Statistical analysis

Free text was recoded into common terminology while cross-checking for entry errors by the two first authors (S.A., J.S.). For binary or categorical variables, the results were reported as counts and proportions. For continuous variables, the results were reported as the mean with standard deviation when normally distributed, or as median with interquartile range when not-normally distributed. Normality was assessed by visual inspection of histograms. Differences in baseline characteristics were assessed using the chi-square test or Fisher's exact test for categorical variables and the Mann–Whitney U test, or Student's *t* test for continuous variables when appropriate.

The primary analysis included descriptive statistics. The preferred ASA score within a case was defined as the ASA score that was chosen by the highest percentage of participants. To determine differences among regions, and among anaesthesiologists and surgeons, chi-square and Fisher's exact tests were used as appropriate. To determine the clinical relevance, the absolute largest difference (ALD) within the preferred scores among regions and anaesthesiologists and surgeons was evaluated. A ≥10% difference among raters was considered clinically relevant. The inter-rater variability was calculated using Light’s Kappa (Kappa for more than two raters) and defined as the K-value. Inter-rater variability describes the degree of agreement among independent observers who rate or assess the same concept. Interpretation of the K-value was based on the Kappa value interpretation as determined by Landis and Koch: poor (≤0), slight (0.01–0.20), fair (0.21–0.40), moderate (0.41–0.60), substantial (0.61–0.80), or perfect agreement (0.81–1.00)^15^. A two-tailed *P* of <0.05 was considered statistically significant. Results were analysed using RStudio (version 4.2.1).

## Results

Overall, 17 of 133 invited anaesthesiology societies (12.8%) and 9 of 10 invited surgical societies (90%) disseminated the survey to their members, including the ASA. In total, 1729 participants responded. Following exclusion of incomplete responses, 1283 participants (74.2%) were included in the analytic cohort. Respondents worked in 55 different countries: 601 in North America (47%), 423 in Europe (33%), 150 in the Asia-Pacific (12%), 96 in South America (7%), and 11 in Africa (0.9%). Respondents included 1073 anaesthesiologists (84%) among whom the majority worked in a general anaesthesiology unit with an interest in HPB (*n* = 588; 46%); among the 210 surgeons (16%), the majority worked in a dedicated HPB unit without liver transplant surgery (*n* = 89; 42%). Baseline characteristics are reported in *Table 1*.

**Table 1:** Baseline characteristics

	Total (*n* = 1283)	Anaesthesiologist (*n* = 1073)	Surgeon (*n* = 210)	*P*
**Sex**				**<0.001**
Female	409 (32)	384 (36)	25 (12)	
Male	867 (68)	683 (64)	184 (88)	
Non-binary/third gender	7 (0.5)	6 (0.6)	1 (0.5)	
Age, years, median (i.q.r.)	48.0 (40.0–57.0)	48.0 (40.0–58.0)	49.5 (41.0–56.0)	0.724
**Continent**				**<0.001**
** **Africa	11 (0.8)	10 (0.9)	1 (0.5)	
Asia-Pacific	150 (11.7)	105 (9.8)	45 (21)	
Europe	423 (32.9)	280 (26)	143 (68)	
North America	601 (46.8)	586 (55)	15 (7)	
South America	96 (7.5)	91 (8.5)	5 (2)	
Unknown	2 (0.1)	1 (<0.1)	1 (0.5)	
**Hospital type**				**<0.001**
Academic (central)	685 (53.4)	509 (47)	176 (84)	
Non-academic, teaching	311 (24.2)	279 (26)	8 (4)	
Non-academic, non-teaching	287 (22.4)	285 (27)	26 (12)	
Years registered as specialist, median (i.q.r.)	17.0 (9.0–26.0)	17.0 (9.0–26.0)	19.5 (10.0–25.0)	0.535
**Level of HPB surgery expertise**				**<0.001**
Dedicated HPB unit, no transplant	273 (21.3)	184 (17)	89 (42)	
Dedicated HPB unit, transplant	343 (26.7)	264 (25)	79 (38)	
General surgery, interest HPB	420 (32.7)	380 (35)	40 (19)	
No HPB surgery	233 (18.1)	231 (22)	2 (1)	
Unknown	14 (1.1)	14 (1.3)	0 (0)	
**Level of HPB anaesthesiology expertise**				**<0.001**
Dedicated HPB unit, no transplant	116 (9.0)	87 (8)	29 (14)	
Dedicated HPB unit, transplant	333 (25.9)	259 (24)	74 (35)	
General anaesthesiology, interest HPB	588 (46)	493 (46)	95 (45)	
No HPB surgeries performed	225 (17.5)	220 (21)	5 (2)	
Unknown	21 (1.6)	14 (1.3)	7 (3)	

Values are *n* (%) unless indicated otherwise. Bold numbers indicate statistical significance. i.q.r., interquartile range; HPB, hepato-pancreato-biliary.

### Practical use

**Fig. 1:**
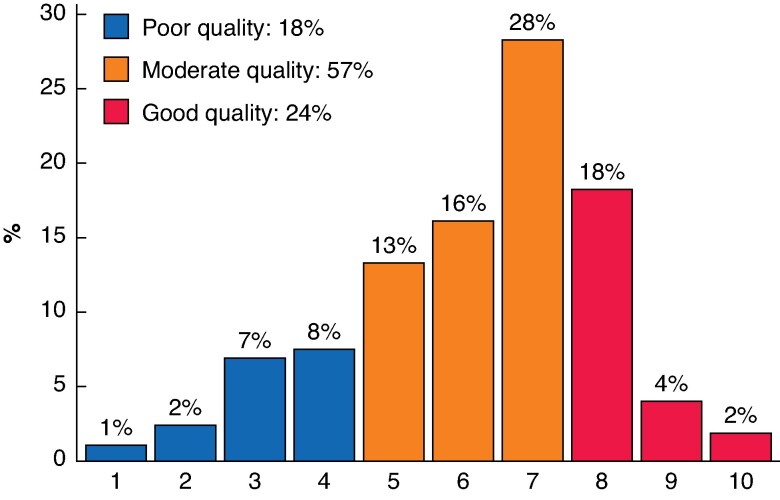
Quality of American Society of Anesthesiologists (ASA) classification according to respondents Score 1–10 for quality of the ASA classification. 1: I consider ASA a poor classification system, non-objective with very poor interobserver agreement, 10: I consider ASA an excellent classification system, highly objective and excellent interobserver agreement.

**Fig. 2:**
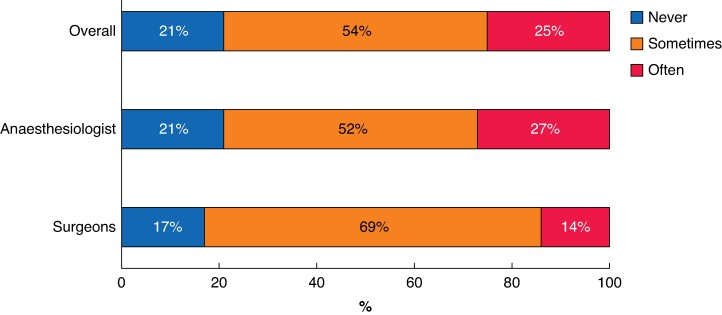
Does the American Society of Anesthesiologists (ASA) score assigned to a patient change your perioperative strategy?

Most respondents used the ASA classification (*n* = 1003, 78%), and some of these used additional prediction tools to determine patients’ perioperative risks (*n* = 255; 20%). When queried about their perception of the ASA classification as an objective system, most respondents (*n* = 362, 28%) provided a rating of 7/10 (*Fig. 1*). The median score of all respondents was 6.0 (i.q.r. 5.0–7.0, *Fig. 1*), which was higher among anaesthesiologists *versus* surgeons (7.0 *versus* 6.0, *P* < 0.001). Seventy-five percent (*n* = 1019) of respondents reported that patient ASA classification could lead to a change in the perioperative strategy (anaesthesiologists, 73% (*n* = 845) *versus* surgeons, 86% (*n* = 174; *Fig. 2*).

A variance in the additional prediction tools employed was observed (full list: *Supplementary Material S3*). The most frequently used scores were the STOP-BANG score (Sleep Apnea)^16^, Revised Cardiac Risk Index (Lee Criteria)^17^, MET score (functional capacity)^18^, Frailty Index^19^, and the ACS NSQIP surgical risk score^20^. In addition, clinical assessment tools such as medical history and functional tests were used. Among the 2% (*n* = 57) of respondents who did not use the ASA score, most reported that they solely used a patient’s medical history (*Supplementary Material S4*). Anaesthesiology departments scored the ASA classification (*n* = 1272, 99%), with the assessment performed by a medical specialist (*n* = 943, 73%), a resident (*n* = 213, 17%), a specialized nurse (*n* = 23, 2%), or all the above equally (*n* = 104, 8%). Among respondents involved in research (*n* = 762), the majority ‘always’ used the ASA classification within research (*n* = 369, 48%), followed by ‘often’ (*n* = 212, 28%), ‘sometimes’ (*n* = 147, 29%), and ‘never’ (*n* = 34, 4%).

### Variables considered for ASA classification

**Fig. 3:**
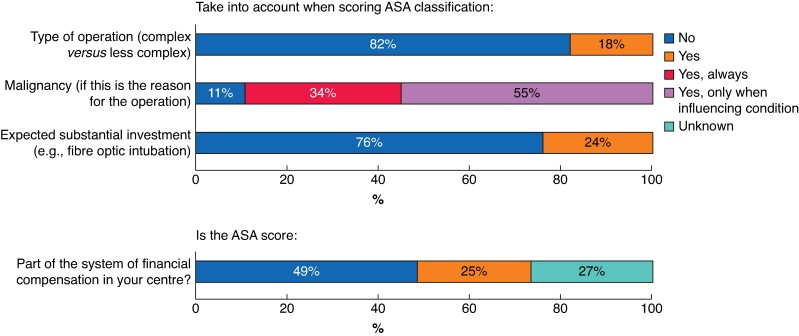
Considerations made when classifying patients according to American Society of Anesthesiologists (ASA) score

**Fig. 4:**
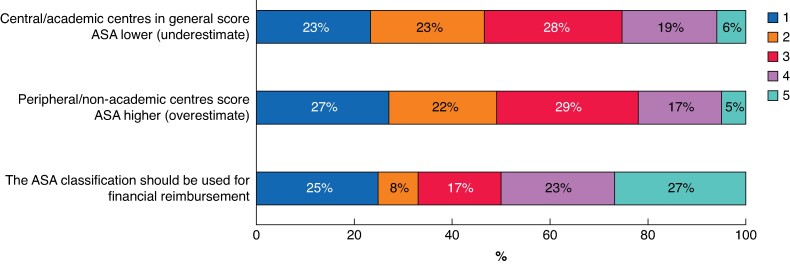
**Considerations made in the American Society of Anesthesiologists (ASA) scoring process** Rated on a scale of 1–5, with 1 strongly disagree, and 5 strongly agree.

When classifying patients according to ASA, most respondents (*n* = 1054, 82%) did not take the type of operation (complex *versus* less complex) and the complexity of anaesthetic management (for example fibre optic intubation, *n* = 981, 76%) into account. In contrast, nearly all respondents (*n* = 1136, 89%) considered cancer as a co-morbidity within scoring, particularly when the clinical condition of the patient was impaired (*Fig. 3*). A minority of the participants believed that academic centres score ASA lower (*n* = 329, 25%) and non-academic centres score ASA higher (*n* = 280, 22%) (*Fig. 4*). This was slightly more prevalent in the 499 participants working in an anaesthesiology-dedicated HPB unit (*n* = 145, 32% believe academic centres score lower, and *n* = 126, 28% believe peripheral centres score higher, *P* < 0.001) than in 588 participants working in a general anaesthesiology unit (*n* = 134, 23% believe academic centres score lower, and *n* = 115, 20% believe peripheral centres score higher, *P* < 0.001). Half the participants stated that the ASA classification should be used for financial reimbursement from health insurance companies (*Fig. 4*). No consensus was observed on the highest percentage of disagreement among ASA score that would be acceptable in clinical practice (for example ‘less than 10%’ *n* = 507 (40%) *versus* ‘less than 20%’ *n* = 404 (30%) *versus* ‘less than 5%’ *n* = 269 (21%)). The five most-reported suggestions to reduce undesired variability in the ASA classification and reflect the true perioperative risk were as follows: take type of operation into account (*n* = 145, 12.5%), more extensive ASA definition (*n* = 137, 11.9%), more examples (*n* = 94, 8.1%), take performance status into account (*n* = 77, 6.7%), and a further ASA subdivision (*n* = 73, 6.3%) (*Table 2*). Among all respondents, 1156 (90%) mentioned at least one factor that should be added to the definition of ASA classification (*Supplementary Material S5*).

**Table 2:** Top 10 suggestions to improve the inter-rater variability of the ASA classification

Suggestions to add to the ASA classification	No. of times reported*
1. Include type of procedure	145
2. More extensive definition	137
3. Provide more examples	94
4. Include performance status (functional status)	77
5. Further subdivision	73
6. Include frailty	70
7. Include age	49
8. Include more co-morbidities	40
9. Other scores	38
10. Include BMI/weight	34

*Multiple suggestions can be made by one respondent. ASA, American Society of Anesthesiologists; BMI, body mass index.

### Clinical case-vignettes

The most commonly chosen option across all case-vignettes was ASA III or IV (*Table 2*). Within the pancreatoduodenectomy case-vignettes, the most commonly chosen option was ASA II. However, it became ASA III when either the patient had coronary artery bypass grafting (CABG) 2 years prior (case 2) or the patient's condition did not recover after biliary stenting (case 3). Changing the age (83 instead of 55 years old, case 4) did not make a difference, as the preferred ASA score remained II. For all case-vignettes that included a patient undergoing an extended left hemihepatectomy, the preferred ASA score was III, irrespective of diabetes mellitus, robotic instead of open procedure, or a benign diagnosis.

The inter-rater variability was fair/moderate within all clinical cases (Kappa range 0.26–0.42, *Table 3*). Six Kappa values all corresponded to ‘fair agreement’, and in case 2 ‘moderate agreement’ was reached (Kappa 0.42). The case in which moderate agreement was reached described a patient who underwent pancreatoduodenectomy and had severe co-morbidities (CABG 2 years prior).

**Table 3:** Clinical cases and inter-rater variability

Type of surgery	Case	Co-morbidities and clinical condition	ASA score		Agreement*
Pancreatoduodenectomy	1	Diagnosis: distal bile duct carcinoma 55 years old, BMI 30 kg/m^2^ Biliary stasis and a general decline in condition (since 3 months) After stenting recovered condition No other medical history	ASA 1	71 (6)	Fair agreement (K = 0.31)
			ASA 2	**730 (57)**	
			ASA 3	445 (35)	
			ASA 4	35 (3)	
			ASA 5	2 (0)	
	2	Diagnosis: distal bile duct carcinoma 55 years old, BMI 30 kg/m^2^ Biliary stasis and a general decline in condition (since 3 months) After stenting recovered condition **CABG 2 years ago, no cardiac complaints, moderate left and right ventricle function**	ASA 1	3 (0)	Moderate agreement (K = 0.42)
			ASA 2	283 (22)	
			ASA 3	**892 (70)**	
			ASA 4	99 (8)	
			ASA 5	6 (0)	
	3	Diagnosis: distal bile duct carcinoma 55 years old, BMI 30 kg/m^2^ Biliary stasis and a general decline in condition (since 3 months) **After stenting the condition did recover** **+ kidney failure stage 3 (eGFR 40)**	ASA 1	1 (0)	Fair agreement (K = 0.38)
			ASA 2	32 (2)	
			ASA 3	**803 (63)**	
			ASA 4	435 (34)	
			ASA 5	12 (1)	
	4	Diagnosis: distal bile duct carcinoma **83 years old**, BMI 30 kg/m^2^ Biliary stasis and a general decline in condition (since 3 months) After stenting recovered condition No other medical history	ASA 1	39 (3)	Fair agreement (K = 0.26)
			ASA 2	**589 (46)**	
			ASA 3	561 (44)	
			ASA 4	85 (7)	
			ASA 5	9 (1)	
Hemihepatectomy	5	Diagnosis: 1 metachronous colorectal liver metastasis 73 years old, open procedure No neoadjuvant therapy, no other liver disease Preoperative work-up aorta valve stenosis (AVA 1.2 cm^2^) Runs 5 miles 2× a week, no other medical history	ASA 1	47 (4)	Fair agreement (0.26)
			ASA 2	544 (42)	
			ASA 3	**601 (47)**	
			ASA 4	87 (7)	
			ASA 5	4 (0)	
	6	Diagnosis: 1 metachronous colorectal liver metastasis 73 years old, open procedure No neoadjuvant therapy, no other liver disease Preoperative work-up aorta valve stenosis (AVA 1.2 cm^2^) Runs 5 miles 2× a week, **insulin-dependent diabetes mellitus**	ASA 1	3 (0)	Fair agreement (K = 0.35)
			ASA 2	334 (26)	
			ASA 3	**810 (63)**	
			ASA 4	133 (10)	
			ASA 5	3 (0)	
	7	Diagnosis: 1 metachronous colorectal liver metastasis 73 years old, **robotic procedure** No neoadjuvant therapy, no other liver disease Preoperative work-up aorta valve stenosis (AVA 1.2 cm^2^) Runs 5 miles 2× a week, no other medical history	ASA 1	9 (1)	Fair agreement (K = 0.33)
			ASA 2	385 (30)	
			ASA 3	**773 (60)**	
			ASA 4	11 (9)	
			ASA 5	5 (0)	
	8	**Diagnosis: hepatocellular adenoma** 73 years old, open procedure No neoadjuvant therapy, no other liver disease Preoperative work-up aorta valve stenosis (AVA 1.2 cm^2^) Runs 5 miles 2× a week, no other medical history	ASA 1	48 (4)	Fair agreement (K = 0.27)
			ASA 2	565 (44)	
			ASA 3	**594 (46)**	
			ASA 4	72 (6)	
			ASA 5	4 (0)	

Values are *n* (%). Bold text indicates change in the clinical condition per case and the preferred American Society of Anesthesiologists (ASA) score. *Based on the Kappa value interpretation determined by Landis and Koch^15^. BMI, body mass index.

A clinically relevant difference (≥10%) in ASA score among regions was noted in all case-vignettes (ALD range: 12–36%). Among North American respondents, in 8/8 (100%) cases the most commonly chosen ASA score was III. ASA score III was most commonly chosen in 3/8 (38%) cases in the Asia-Pacific, 4/8 (50%) in Europe, 4/8 (50%) in South America (50%), and 7/8 (88%) in Africa; the rest of the cases were scored ASA II (*Supplementary Material S6*). Significant inter-rater variability within regions was detected as well (*Supplementary Material S8*). A clinically relevant difference among anaesthesiologists and surgeons was also noted for 6/8 cases (ALD range: 23–30%, *Supplementary Material S7*). This finding was noted in all four of the hemihepatectomy case-vignettes. Surgeons scored lower (ASA II instead of ASA III).

## Discussion

This international survey and case-vignette study identified a considerable inter-rater variability regarding the ASA classification of patients undergoing HPB surgery. Most respondents used the ASA classification (78%), among whom 79% adapted their perioperative strategy according to its results. Over 80% of respondents stated that they would take the underlying disease (for example cancer) into account for ASA scoring, but this barely changed the ASA score within the case-vignettes (1% difference in most reported score). Only 11% stated that they would take the type of operation into account (for example complex surgery); nevertheless, this did change the preferred score in the case-vignettes (13% difference).

The inter-rater variability in ASA classification among patients undergoing common (less-complex) surgical procedures has been confirmed in surveys from Finland, Italy, the United Kingdom, and the United States^2–5^. The Kappa values reported in these studies, ranging from 0.21 to 0.40, were comparable to those in the present international study^2,4^. In addition to the heterogeneity in ASA scoring in hypothetical cases, substantial variation in the classification of patients exists in daily clinical practice^21^. Suggestions have been made to improve the ASA classification, such as adding institutional-specific or ASA-approved examples to the classification, which have been proven to decrease variability in scoring^22,23^. Examples were added to paediatric and obstetric patients specifically, as the ASA score is more variable for these subgroups^24,25^. These additions are not yet implemented for patients undergoing HPB surgery, even though considerable differences exist in this patient group. HPB surgery is highly complex surgery, and often includes frail patients who typically undergo surgery for cancer. The benefits from surgery must be carefully weighted relative to the perioperative risk, which is often determined by various risk factors including the ASA score.

Within all case-vignettes included in this survey, the most commonly chosen ASA score was either ASA II or ASA III, whereas ASA I and ASA IV–V were almost never used. If certain values are barely used, the Kappa score can get less reliable, a phenomenon known as the ‘Kappa paradox’^26^. Within the case-vignettes, the decision to use ASA II or III seemed often inconsistent. For example, in the fourth case-vignette ASA II was scored 46%, and ASA III 44%. This distinction is most important, as many clinical studies that use ASA as a case-mix adjustment make a subdivision between patients scored either ASA I–II or ASA III. Suggestions mentioned within the survey to decrease inter-rater variability included the creation of a more extensive definition (137 respondents) or a further subdivision (77 respondents), which could better differentiate ASA II from ASA III. However, it should be taken into account that the ASA classification was designed as a pragmatic bedside tool and has to stay easy-to-use in daily clinical practice^27^.

Adding examples to the ASA definition may decrease inter-rater variability, whereas the classification remains feasible in daily clinical practice^24,25^. It seems that the extent of surgery and the surgical approach is already considered when scoring the ASA classification (60% of the participants classified case 7 robotic hemihepatectomy as ASA III, and this decreased to 47% when an open hemihepatectomy was performed). Benign *versus* malignant disease does not seem to influence the ASA score (case 5 colorectal liver metastasis: 47% ASA III; case 8 hepatocellular adenoma: 46% ASA III), even though the vast majority of respondents (89%) stated that a malignancy should be considered for ASA classification. These data illustrate the lack of consensus and confirm the need for an extended definition or addition of examples to the classification (for example including remarks on the impact of the type of surgery, malignant *versus* benign disease).

ASA scoring differs among regions, which could be due to financial incentives associated with ASA classification in some countries, given that 50% of the participants report that the ASA score influences financial compensation in their country. Convincing evidence on this topic is scarce. One study from the USA examined variation in ASA scores relative to insurance eligibility^28^. This study reported no up-coding of ASA score with changes in payer incentives.

The Simplified Acute Physiology Score II (SAPS II) was reported to have the highest power to predict mortality, but it is rarely used in clinical practice^29^. The Charlson Co-morbidity Index (CCI) has been reported to have lower discriminative power to predict mortality, but might be more suitable for case-mix adjustment within research, as less heterogeneity could be expected (especially among countries) due to its more explicit criteria^29^. The ASA classification is easy to use and associated with multiple postoperative outcomes including postoperative morbidity and mortality^30^. Improvements made in the ASA classification, such as the addition of examples, have been proven to reduce inter-rater variability. Until this has been achieved, especially when comparing outcomes among countries, researchers should consider using more comprehensive measures such as the CCI for case-mix adjustment.

These results should be interpreted in light of some limitations. Among 133 (inter)national anaesthesiology societies, only 17 (12%) disseminated the survey. Unfortunately, the exact response rate is unknown, as the number of members within the different societies could not be shared. Most respondents were from Europe/North America (80%), limiting the generalization. Answers to the questions on ‘the considerations made during the ASA classification process’ were very heterogeneous, making it hard to draw any firm conclusion. The heterogeneity in the answers does represent the current lack of consensus on the considerations made in the decision-making of the ASA score (that is, which parameters should be considered). Almost all surgeons who responded performed HPB surgery (99%), but only 78% of all anaesthesiologists worked with HPB patients (of which the minority worked in a dedicated HPB unit: 32%), which might have influenced the results. Future research should focus on assessing the clinical and socioeconomic impact of the observed variability, achieving consensus on how the ASA classification should be used (for example take indication for surgery and type of surgery into account or not) to improve consistent use and interpretation of the classification, as well as seeking ways to adjust ASA to make it more applicable to the clinical setting (for example extending the main classification, or adding HPB surgery-specific examples).

This study demonstrated variability in interpretation and use of the ASA classification by a large cohort of international anaesthesiologists and surgeons caring for HPB surgical patients. A need exists for an improved, more objective, ASA classification to facilitate harmonized clinical decision-making and minimize inappropriate conclusions from studies employing incorrect ASA classifications. This could be achieved by adding examples to the classification and raising awareness on the topic. Until then, more objective measurements should be considered for case-mix adjustment within research in patients undergoing HPB surgery.

## Supplementary Material

zrae162_Supplementary_Data

## Data Availability

Data can be made available upon reasonable request.
