# Supplementary material for: Inter-rater variability for the American Society of Anesthesiologists classification in patients undergoing hepato-pancreato-biliary surgery (MILESTONE-2): international survey among surgeons and anaesthesiologists
Source: BJS Open. 2025 Feb 28;9(1):zrae162. doi: 10.1093/bjsopen/zrae162 (PMC11879015; doi:10.1093/bjsopen/zrae162)
Supplement: zrae162_Supplementary_Data [file zrae162_supplementary_data.docx]

**Interrater variability for the ASA classification in patients undergoing hepato-pancreato-biliary surgery (MILESTONE-2): international survey among surgeons and anesthesiologists**

Simone Augustinus MD^1,2^*, Jasper P. Sijberden MD^1-3^*, Matthanja Bieze MD PhD^4,5^, Vandana Agarwal MD FRCA^6^, Luca A. Aldrighetti MD PhD^7^, Adnan Alseidi MD EdM^8^, Francisco C. Bonofiglio MD PhD^9^, Kevin C.P. Conlon MD PhD MBA^11^, Katia Donadello MD^12^, Joris Erdmann MD PhD^1,2^, Cristina Ferrone MD PhD^13^, Michael Guertin MD MBA^14^, Ronald Harter MD^14^, Maria E. Franceschetti MD^15^, Guiseppe K. Fusai MD PhD^16^, Bas Groot Koerkamp MD MSc PhD^17^, Thilo Hackert MD PhD^18^,

Jin-Young Jang MD PhD^19^, Thomas Kander MD PhD^20^, Tobias Keck MD PhD MBA^21^, Dominik

Krzanicki MB ChB FRCA^22^, Ho-Jin Lee MD PhD^23^, Keith Lewis MD PhD^24^, Giuseppe Natalini MD^15^,

Carla Nau MD PhD^25^, Timothy M. Pawlik MD PhD MPH^26^, Henry A. Pitt MD^27^, Rafaella Reineke MD PhD^28^, Roberto Salvia MD PhD^29^, Eduardo de Santibanes MD PhD^30^, Shailesh V. Shrikhande MD PhD^31^,

Martin Smith MD PhD^32^, Attila Szijarto MD PhD^33^, Bobby Tingstedt MD PhD^34^, Alice C. Wei MD MSc^35^,

John Windsor MD PhD^36^, Mohammed Abu Hilal MD PhD^3^#, Manuel Pardo MD^37^#,

Markus W. Hollmann MD PhD^4^#, Marc G. Besselink MD MSc PhD^1,2^# for the MILESTONE study group.

*Shared first authorship, #shared senior authorship.

^1^Amsterdam UMC, location University of Amsterdam, Department of Surgery, Amsterdam, the Netherlands; ^2^Cancer Center Amsterdam, Amsterdam, the Netherlands; ^3^Department of Surgery, Fondazione Poliambulanza Istituto Ospedaliero, Brescia, Italy; ^4^Amsterdam UMC, location University of Amsterdam, Department of Anesthesiology, Amsterdam, the Netherlands; ^5^Department of Anesthesiology and Pain Management, Toronto General Hospital, University of Toronto, Toronto, Ontario, Canada; ^6^Department of Anesthesia, Critical Care and Pain, Tata Memorial Hospital, Homi Bhabha National Institute, Mumbai, Maharashtra, India; ^7^Hepatobiliary Surgery Division, Ospedale San Raffaele, Milano, Italy; ^8^Division of Hepatopancreatobiliary and Endocrine Surgery, University of California, San Francisco, CA, USA; ^9^Department of Anesthesiology, Hospital Italiano de Buenos Aires, Buenos Aires, Argentina; ^11^Department of Surgery, Trinity College Dublin, Tallaght University Hospital, Dublin, Ireland; ^12^Department of Anesthesia and Intensive Care B, DSCOMI, University of Verona, University Hospital Integrated Trust of Verona, Verona, Italy; ^13^Department of Surgery, Massachusetts general Hospital, Harvard Medical School, Boston, Massachusetts, USA; ^14^Department of Anesthesiology, The Ohio State University Wexner Medical Center & College of Medicine, Ohio, USA; ^15^Department of Anesthesia and Intensive Care, Istituto Fondazione Poliambulanza, Brescia, Italy; ^16^Hepatobiliary Surgery and Liver Transplantation Unit, Royal Free Hospital, London, United Kingdom; ^17^Department of Surgery, Erasmus MC, University Medical Center Rotterdam, Rotterdam, The Netherlands; ^18^Department of General, Visceral and Thoracic Surgery, University Hospital Hamburg-Eppendorf, Germany; ^19^Department of Surgery and Cancer Research Institute, Seoul National University College of Medicine, Seoul, South Korea; ^20^Department of Intensive and Perioperative Care, Skåne University Hospital, Lund and Lund University, Sweden; ^21^DGAV StuDoQ|Pancreas and Clinic of Surgery, UKSH Campus Lübeck, Germany; ^22^Department of Anaesthesia, Royal Free Hospital, London, United Kingdom; ^23^Department of Anesthesiology and Pain medicine, Seoul National University Hospital, Seoul, South Korea; ^24^Department of Anesthesiology and Perioperative Medicine, Rutgers Robert Wood Johnson Medical School, New Brunswick, NJ, USA; ^25^Department of Anaesthesiology and Intensive Care, University Medical Centre Schleswig-Holstein, Campus Lübeck, Lübeck, Germany.^26^Department of Surgery, The Ohio State University Wexner Medical Center, Columbus, Ohio, USA; ^27^Department of Surgery, Rutgers Cancer Institute of New Jersey, New Brunswick, New Jersey, USA; ^28^Department of Anesthesiology and Intensive Care, IRCCS San Raffaele Scientific Institute, Milan, Italy; ^29^General and Pancreatic Surgery Department, Pancreas Institute, University and Hospital Trust of Verona, Verona, Italy; ^30^Department of Surgery, Hospital Italiano, University of Buenos Aires, Buenos Aires, Argentina; ^31^Department of GI and HPB Surgery, Tata Memorial Centre, Homi Bhabha National Institute, Mumbai, India; ^32^Department of Surgery, Faculty of Health Sciences, School of Clinical Medicine, University of the Witwatersrand, Johannesburg, South Africa; ^33^Department of Anesthesiology, I. sz. Sebészeti Klinika, Semmelweis Egyetem, Budapest; ^34^Department of Surgery, Clinical Sciences Lund, Lund University, Skåne University Hospital, Lund, Sweden; ^35^Hepatopancreatobiliary Service, Department of Surgery, Memorial Sloan Kettering Cancer Center, New York, USA; ^36^HBP/Upper GI Unit, Auckland City Hospital/Department of Surgery, University of Auckland, New Zealand; ^37^Department of Anesthesia and Perioperative Care, University of California, San Francisco, CA, USA

**Corresponding author.**

Marc G. Besselink, MD

Department of Surgery, Amsterdam UMC

University of Amsterdam, Cancer Center Amsterdam

De Boelelaan 1117 (ZH-7F), 1081 HV Amsterdam, the Netherlands

E-mail: m.g.besselink@amsterdamUMC.nl (during review: [s.augustinus@amsterdamUMC.nl](mailto:s.augustinus@amsterdamUMC.nl))

**ORCID ID:** 0000-0003-2650-9350; **Twitter**: @MarcBesselink

**Supplementary Materials - Index**

| **Supplementary Methods** |  |
| --- | --- |
| Supplementary material 1. Invited societies | *pag. 3* |
| Supplementary material 2. Survey and clinical cases | *pag. 11* |
| **Supplementary Results** |  |
| Supplementary material 3. Additional assessment tools used (besides the ASA classification) | *pag. 17* |
| Supplementary material 4. Pre-operative assessment tool(s) when the ASA score is not used | *pag. 20* |
| Supplementary material 5. Beyond top 10 suggestions to add to the ASA classification | *pag. 21* |
| **Supplementary Appendixes** |  |
| X | *pag. 23* |
| **Supplementary Figures and Tables** |  |
| Supplementary material 6. Differences in ASA scores among regions | *pag. 24* |
| Supplementary material 7. Interrater variability within continents | *pag. 25* |
| Supplementary material 8. Difference in ASA score among surgeons and anesthesiologists |  |
| **References** | *pag. 27* |
|  |  |

**Supplementary Methods**

**Supplementary material 1.** Invited societies

| **Anesthesiology** | | **Surgical / HPB society** | |
| --- | --- | --- | --- |
| **Invited** | **Agreed** | **Invited** | **Agreed** |
| Liver Intensive Care Groups of Europe (LICAGE) | Yes | International Hepato-Pancreato-Biliary Association (IHPBA) | Yes |
| American Society of Anesthesiologists (ASA) | Yes | European-African Hepato-Pancreato-Biliary Association (EAHPBA) | Yes |
| Albanian Society of Anesthesiologists & Intensivists (ASAI) | **-** | Americas Hepato-Pancreato-Biliary Association (AHPBA) | Yes |
| Albanian Society of Anesthesiologists & Intensivists (ASAI) | **-** | Asia-Pacific Pancreato-Biliary Association (AP-HPBA) | Yes |
| Australian Society of Anaesthetists | **-** | International Laparoscopic Liver Society (ILLS) | Yes |
| Österreichische Gesellschaft für Anasthesiologie, Reanimation und Intensivmedizin | **-** | European Consortium on Minimally Invasive Pancreatic Surgery (E-MIPS) | Yes |
| Bangladesh Society of Anaesthesiologists, Critical Care and Pain Physicians | **-** | European Minimally Invasive Liver Surgical Group (E-MILS) | Yes |
| Belgian Society of Anesthesiology, Resuscitation, Perioperative Medicine and Pain Management | **-** | Association of Upper Gastrointestinal Surgeons of Great Britain and Ireland (AUGIS) | - |
| Societé d'Anesthesie-Reanimation du Benin | **-** | Dutch Pancreatic Cancer Group (DPCG) | Yes |
| Sociedad Boliviana de Anestesiología Reanimación y Dolor | **-** | Turkish HPB society | Yes |
| Association of Anesthesiologists and Reanimathologists of FBiH | **-** |  |  |
| Botswana Society of Anaesthetists | **-** |  |  |
| Brazilian Society of Anesthesiology (SBA Sociedade Brasileira de Anestesiologia) | Yes |  |  |
| Society of Anaesthesiologists, Brunei Darussalam | **-** |  |  |
| Bulgarian Society of Anesthesiology and Intensive Care | **-** |  |  |
| Société d'Anesthésie, de Réanimation et de Médecine d'Urgence du Burkina | **-** |  |  |
| Agora des Techniciens Supérieurs Anesthésistes Réanimateurs pour la Promotion de la Santé (ATSARPS) | **-** |  |  |
| Cambodia Society of Anaesthetists | **-** |  |  |
| Société Camerounaise d’Anesthésie Réanimation (S.C.A.R) | **-** |  |  |
| Canadian Anesthesiologists' Society | Yes |  |  |
| Sociedad de Anestesiología de Chile | **-** |  |  |
| Chinese Society of Anesthesiology | **-** |  |  |
| Taiwan Society of Anaesthesiologists | **-** |  |  |
| Sociedad Colombiana de Anestesiología y Reanimación (SCARE) | **-** |  |  |
| Société Congolaise d’Anesthésie Réanimation Urgences | **-** |  |  |
| Anaesthesiology & Intensive Care Society of Democratic Republic of Congo | **-** |  |  |
| Asociación de Médicos Anestesiólogos de Costa Rica | **-** |  |  |
| Société Ivoirienne d'Anesthésie-Réanimation | **-** |  |  |
| Croatian Association of Anaesthesiology and Intensive Care Medicine | **-** |  |  |
| Sociedad Cubana de Anestesiología y Reanimación | **-** |  |  |
| Anaesthesiology Society of Cyprus | **-** |  |  |
| Czech Society of Anaesthesiology and Intensive Care Medicine | **-** |  |  |
| Danish Society of Anaesthesiology and Intensive Care Medicine | **-** |  |  |
| Sociedad Dominicana de Anestesiología | **-** |  |  |
| Sociedad Ecuatoriana de Anestesiología | **-** |  |  |
| Sociedad Ecuatoriana de Anestesiología | **-** |  |  |
| Asociación de Médicos Anestesiólogos de el Salvador | **-** |  |  |
| Estonian Society of Anaesthesiologists | Yes |  |  |
| Eswatini Society of Anaesthetists | **-** |  |  |
| Ethiopian Society of Anaesthesiologists | **-** |  |  |
| Finnish Society of Anaesthesiologists | **-** |  |  |
| Société Française d'Anesthésie et de Réanimation | Yes |  |  |
| Societe Gabonaise d'Analgesie, Anesthesie, Reanimation et de Medecin | **-** |  |  |
| Georgian Society of Anesthesiology and Critical Care Medicine | - |  |  |
| Deutsche Gesellschaft für Anaesthesiologie und Intensivmedizin e.V. (DGAI) | Yes |  |  |
| Ghana Anaesthetist Society | - |  |  |
| Hellenic Society of Anaesthesiology | Yes |  |  |
| Asociacion Guatemalteca de Anestesiologia, Reanimación y Tratamiento del Dolor | **-** |  |  |
| Guyana Anesthesiologist Society | **-** |  |  |
| Société Haitienne d'Anesthésiologie | **-** |  |  |
| Sociedad Hondureña De Anestesiologia, Reanimacion Y Dolor (SHARD) | **-** |  |  |
| The Society of Anaesthetists of Hong Kong | **-** |  |  |
| Hungarian Society of Anaesthesiology and Intensive Therapy | **-** |  |  |
| Icelandic Society of Anaesthesiology and Intensive Care Medicine | **-** |  |  |
| Indian Society of Anaesthesiologists | Yes |  |  |
| Indonesian Society of Anesthesiologists and Intensive Therapy | **-** |  |  |
| Iranian Society of Anesthesiologists and Critical Care | **-** |  |  |
| Iraqi Society for Anaesthesiology, Intensive Care and Pain Medicine (IQSAN) | **-** |  |  |
| Israel Society of Anesthesiologists | **-** |  |  |
| Società Italiana di Anestesia, Analgesia, Rianimazione e Terapia Intensiva | Yes |  |  |
| Japanese Society of Anesthesiologists | **-** |  |  |
| Jordan Society of Anaesthesia and Intensive Care | **-** |  |  |
| Republican Social Union Federation of Anaesthesiologists & Reanimatiologists | **-** |  |  |
| Kenya Society of Anaesthesiologists | **-** |  |  |
| Korean Society of Anesthesiologists | Yes |  |  |
| Society of Anesthesiology at Intensive Care of Kosova - (SAICK) | **-** |  |  |
| Lao Society of Anesthesiologists | **-** |  |  |
| Association of Anaesthesiologists-Reanimatologists of Latvia | **-** |  |  |
| Lebanese Society of Anesthesiologists | **-** |  |  |
| Libyan Society of Anaesthesia, Intensive Care and Resuscitation (L.S.A.I.R) | **-** |  |  |
| Societe D’Anestesie-Reanimation De Madagascar (SARM) | **-** |  |  |
| Society of Anaesthesiologists Malawi | **-** |  |  |
| Malaysian Society of Anaesthesiologists | **-** |  |  |
| Maldives Anaesthesiologists Association | **-** |  |  |
| Société d'Anesthésie, de Réanimation et de Médicine d'Urgence du Mali | **-** |  |  |
| Association of Anaesthesiologists of Malta (AAM) | **-** |  |  |
| Association of Anaesthesiologists of Mauritius | **-** |  |  |
| Federación Mexicana de Colegios de Anestesiología, A.C. | **-** |  |  |
| Micronesia Anesthesia Society | **-** |  |  |
| Society of Anaesthesia and Reanimatology of the Republic of Moldova | **-** |  |  |
| Mongolian Society of Anesthesiologists | **-** |  |  |
| Morrocan Society of Anesthesia, Analgesia and Critical Care (SMAAR) | **-** |  |  |
| Associação de Anestesiologistas de Moçambique | **-** |  |  |
| Myanmar Society of Anaesthesiologists (Myanmar Medical Association) | **-** |  |  |
| Anaesthesiologists Society of Namibia (ASN) | **-** |  |  |
| Society of Anaesthesiologists of Nepal | **-** |  |  |
| Nederlandse Vereniging voor Anesthesiologie | Yes |  |  |
| New Zealand Society of Anaesthetists | Yes |  |  |
| Associación Nicaragüense de Anestesiología y Reanimación | **-** |  |  |
| Nigerian Society of Anaesthetists | **-** |  |  |
| Macedonia Society of Anaesthesiologists | Yes |  |  |
| Norsk Anestesiologisk Forening |  |  |  |
| Oman Society of Anesthesia & Critical Care | Yes |  |  |
| Pacific Society of Anaesthetists | **-** |  |  |
| Pakistan Society of Anaesthesiologists | **-** |  |  |
| Palestinian Society of Anaesthesia & Intensive Care | **-** |  |  |
| Sociedad Panameña de Anestesiología | **-** |  |  |
| Society of Anaesthetists of Papua New Guinea | **-** |  |  |
| Sociedad Parguaya de Anestesiología | **-** |  |  |
| Sociedad Peruana de Anestesia, Analgesia y Reanimación | **-** |  |  |
| Philippine Society of Anaesthesiologists | **-** |  |  |
| Polish Society of Anaethesiology and Intensive Therapy | **-** |  |  |
| Sociedade Portuguesa de Anetesiologia | **-** |  |  |
| Association of Anaesthesiologists & Reanimatologists of the Republic of Srpska | **-** |  |  |
| The Romanian Society of Anaesthesia - Intensive Care | **-** |  |  |
| Federation of Anaesthesiologists and Reanimatologists (FAR) | **-** |  |  |
| Rwanda Society of Anesthesiologists | **-** |  |  |
| Saudi Anaesthesia Society | **-** |  |  |
| Societé Sénégalaise d'Anesthésie-Réanimation et de Médicine d'Urgence | **-** |  |  |
| Serbian Association of Anaesthesiologists and Intensivists (SAAI) | **-** |  |  |
| Singapore Society of Anaesthesiologists | **-** |  |  |
| Slovak Society of Anaesthesiology and Intensive Medicine | **-** |  |  |
| Slovenian Society of Anaesthesiology and Intensive Care Medicine (SSAICM) | **-** |  |  |
| Somali Society of Anaesthesiologists (SOSA) | **-** |  |  |
| South African Society of Anaesthesiologists | Yes |  |  |
| Sociedad Española de Anestesiología, Reanimación y Terapéutica del Dolor | **-** |  |  |
| College of Anaesthesiologists and Intensivists of Sri Lanka | **-** |  |  |
| Sudanese Society of Anaesthesiologists | **-** |  |  |
| Swedish Society for Anaesthesiology and Intensive Care Medicine | Yes |  |  |
| Schweizerische Gesellschaft für Anästhesiologie und Reanimation | **-** |  |  |
| Society of Anaesthesiologists of Tanzania | **-** |  |  |
| Royal College of Anesthesiologists of Thailand | **-** |  |  |
| The Trinidad & Tobago Anaesthetists Association | **-** |  |  |
| Societé Tunisienne d'Anesthésie et de Réanimation | **-** |  |  |
| Turkish Anaesthesiology and Reanimation Society | **-** |  |  |
| Association of Anaesthesiologists of Uganda (AAU) | **-** |  |  |
| Ukranian Society of Anaesthesiologists | **-** |  |  |
| Association of Anaesthetists (United Kingdom and Ireland) | **-** |  |  |
| Sociedad de Anestesiología del Uruguay | **-** |  |  |
| Uzbekistan Society of Anaesthesiology and Intensive Care | **-** |  |  |
| Sociedad Venezolana de Anestesiología | **-** |  |  |
| Vietnam Society of Anaesthesiologists | **-** |  |  |
| Vietnam Society of Anaesthesiologists | **-** |  |  |
| Society of Anaesthetists of Zambia | **-** |  |  |
| Zimbabwe Anaesthetic Association | **-** |  |  |

**Supplementary Material 2.** Survey and clinical cases

Baseline questions: participant

1. In which country do you work

- Country

2. What is your gender?

- Male
- Female

3. What is your age?

- Age

4. What is your medical specialty?

- Anesthesiologist
- Surgeon

5. How many years of experience do you have as a fully licensed medical specialist?

- Years

6. In what type of hospital do you work?

- Academic hospital (central)
- Non-Academic, teaching (peripheral)
- Non-Academic, non-teaching (peripheral)

7. What level of expertise in HPB surgery is present in your hospital?

- Dedicated HPB unit – including liver transplant surgery
- Dedicated HPB unit – excluding liver transplant surgery
- General surgery unit with interest in HPB
- No HPB surgery in my hospital

8. What level of expertise in HPB anesthesiology is present in your hospital?

- Dedicated HPB anesthesiologist – including liver transplant surgery
- Dedicated HPB anesthesiologist – excluding liver transplant surgery
- General anesthesiology unit with interest in HPB
- No HPB surgeries are performed in my hospital.

General questions: ASA classification

9. What is your overall view on the objectiveness and interobserver agreement of the ASA classification?

- 1-10
  *(1: I consider ASA a poor classification system, non-objective with very poor interobserver agreement, 10: I consider ASA an excellent classification system, highly objective and excellent interobserver agreement)*

10. Which specialty, typically determines a patients’ preoperative ASA score in your hospital?

- Anesthesiologist
- Surgeon

11. Within this specialty, who most often, scores a patients’ preoperative ASA score:

- Medical specialist
- Resident
- (Specialized) nurse
- All of the above evaluate the ASA score equally

12. Is there a preoperative assessment protocol in your hospital, and what does it include?

- Yes, it includes the ASA score
- Yes, it includes the ASA score, plus additional assessment tools 🡪 please specify what others
- Yes, it does not include the ASA score 🡪 please specify what it includes
- No there is no protocol
- Unknown

13. Do you use ASA score (as a case-mix factor) in your surgical clinical research?

- Always
- Often
- Sometimes
- Never
- Not applicable (I am not involved in research)

14. Does the ASA score assigned to a patient change your perioperative strategy (e.g., for surgeons the choice of minimally invasive versus open surgery, or for anesthesiologists choose a specialist instead of a resident leading the anesthesiology team during the operation)?

- Often
- Sometimes
- Never

Considerations made in ASA classification process

In the following questions, considerations made in the ASA classification process will be asked. A description of the ASA classification can be found below, for more information and examples, click on this link (https://www.asahq.org/standards-and-guidelines/asa-physical-status-classification-system).

| ASA PS Classification* | Description |
| --- | --- |
| I | A normal healthy patient |
| II | A patient with mild systemic disease |
| III | A patient with severe systemic disease |
| IV | A patient with severe systemic disease that is a constant threat to life |
| V | A moribund patient who is not expected to survive without the operation |
| VI | A declared brain-dead patient whose organs are being removed for donor purposes |

PS = performance score. *The addition of “E” denotes Emergency surgery: (An emergency is defined as existing when delay in treatment of the patient would lead to a significant increase in the threat to life or body part)

Questions:

15. Do you take the type of operation which will be performed (complex versus less complex operation) into account when scoring the ASA classification?

- Yes
- No
- If yes, how;

16. Do you take a malignancy (if this is the reason for the operation) into account when scoring the ASA classification?

- Yes, always
- Yes, only when the malignancy influences the clinical condition (e.g., bad nutritional status)
- No

17. Do you take an expected substantial investment of time and effort for a patient’s care (e.g., fiber optic intubation) into account when deciding which ASA classification to assign a patient?

- Yes
- No

18. Is the ASA classification part of the system of financial compensation in your center/healthcare system?

- Yes
- No
- Unknown

Please read the following statements and rate on a scale of 1-5, with 1 strongly disagree, and 5 strongly agree:

19. In my healthcare system, central/academic centers in general score ASA lower (underestimate), because they are more used to sicker and more complex patients.

- I-V

20. In my healthcare system, peripheral/non-academic centers score ASA higher (overestimate), because they are less familiar with sicker or more complex patients.

- I-V

21. The ASA classification should be used for financial reimbursement from health insurance companies.

- I-V

22. In your personal view, what should be the highest % of disagreement acceptable in clinical practice if 2 anesthetists or 2 surgeons during a certain period independently both judge a group of 100 patients on ASA score?

- <1%
- <5%
- <10%
- <20%
- <40%
- <60%

23. What would be important to add to the ASA classification to reduce variability and better reflect true perioperative risk:

- Open question

Clinical cases

You are presented with eight cases, four patients who will undergo a pancreatoduodenectomy (PD) and four patients who will undergo an extended left hemihepatectomy without biliary reconstruction (i.e., not a Klatskin resection). In each case (1-4 and 5-8) a subsequent single aspect changes (emphasized in bold). Please choose the ASA classification you would assign to this patient.

**Case 1: PD**A 55-year-old female presents with an occluding carcinoma of the distal bile duct for which she will undergo a pancreatoduodenectomy. Three months ago, she presented with biliary stasis and a general decline in condition. After stenting her condition has now recovered to the previous baseline level. Her BMI is 31 and she has no other medical history.

- ASA I-V

**Case 2: PD**

A 55-year-old female presents with an occluding carcinoma of the distal bile duct for which she will undergo a pancreatoduodenectomy. Three months ago, she presented with biliary stasis and a general decline in condition. After stenting her condition has now recovered to the previous baseline level. **She underwent a CABG 2 years ago, with no current cardiac complaints and moderate left and right ventricle function on ultrasound at recent visit**. Her BMI is 31 and she has no other medical history.

- ASA I-V

**Case 3: Pancreatoduodenectomy**

A 55-year-old female presents with an occluding carcinoma of the distal bile duct for which she will undergo a pancreatoduodenectomy**. Three months ago, she presented with biliary stasis and a general decline in condition.** **After stenting, her condition did not recover to the previous baseline level, and she developed stage 3 kidney failure (eGFR 40).** Her BMI is 31 and she has no other medical history.

- ASA I-V

**Case 4: PD**

A **83-year-old** female presents with an occluding carcinoma of the distal bile duct for which she will undergo a pancreatoduodenectomy. Three months ago, she presented with biliary stasis and a general decline in condition. After stenting her condition has now recovered to the previous baseline level. Her BMI is 31 and she has no other medical history.

- ASA I-V

**Case 5: Hemihepatectomy**

A 73-year-old male presents with one metachronic colorectal liver metastasis, received no neoadjuvant therapy, and has no other liver disease. He will undergo an open extended left hemihepatectomy. In the pre-operative work-up an aortic valve stenosis is found (AVA 1,2 cm^2^). He runs 5 miles twice a week, has no other medical history and does not use any medication.

- ASA I-V

**Case 6: Hemihepatectomy**

A 73-year-old male presents with one metachronic colorectal liver metastasis, received no neoadjuvant therapy, and has no other liver disease. He will undergo an open extended left hemihepatectomy. In the pre-operative work-up an aortic valve stenosis is found (AVA 1,2 cm^2^). He has no other medical history besides **insulin dependent diabetes mellitus**; he runs 5 miles twice a week

- ASA I-V

**Case 7: Hemihepatectomy**

A 73-year-old male presents with one metachronic colorectal liver metastasis, received no neoadjuvant therapy, and has no other liver disease. He is scheduled for **a robotic** extended left hemihepatectomy. In the work-up an aortic valve stenosis is found (AVA 1,2 cm^2^). He has no other medical history besides insulin dependent diabetes mellitus; he runs 5 miles twice a week

- ASA I-V

**Case 8: Hemihepatectomy**

A 73-year-old male presents with a **hepatocellular adenoma,** and has no other liver disease. He will undergo an open extended left hemihepatectomy. In the pre-operative work-up an aortic valve stenosis is found (AVA 1,2 cm^2^). He runs 5 miles twice a week, **has no other medical history** and does not use any medication.

- ASA I-V

**Supplementary Results**

**Supplementary Material 3**. Additional assessment tools used (besides the ASA classification)

| **1. Additional scores used** | **Nr. of times reported*** |
| --- | --- |
| Cardiopulmonal |  |
| STOP-BANG score (OSAS) | 39 |
| Lee (cardiac risk) | 37 |
| MET (functional capacity) | 22 |
| Revised Cardiac Risk Index (RCRI) | 16 |
| New York Heart Association (heart failure) | 13 |
| ChadsVasc (chance on stroke) | 8 |
| Ariscat (pulmonal) | 6 |
| GUPTA (postop respiratory failure) | 5 |
| Duke index (functional capacity patients with cardiac disease) |  |
| Goldman cardiac risk index | 2 |
| COPD GOLD classification | 2 |
| GUPTA (myocardial infarction) | 2 |
| Cardiac scores (not specified) | 7 |
| Detsky score (risk cardiovascular complications) | 2 |
| Helios (cardiopulmonal) | 1 |
| NoSas (OSAS) | 1 |
|  |  |
| Liver |  |
| Child-Pugh score (cirrhosis mortality) | 12 |
| MELD (liver) | 11 |
| VOCAL -Penn (cirrhosis surgical risk score) | 1 |
|  |  |
| Kidney | 1 |
| KDGIO (acute kidney injury staging) | 1 |
|  |  |
| Intubation |  |
| Mallampati (ease of intubation) | 13 |
| Intubation score (not specified) | 5 |
| ULBT score (ease of intubation) | 2 |
| Cormack/Lehane score (ease of intubation) | 2 |
| Arné (intubation score) | 1 |
| Difficult face mask ventilation score | 1 |
|  |  |
| Combined scores |  |
| Frailty score | 23 |
| ACS NSQIP | 21 |
| ECOG / WHO (performance status) | 12 |
| P-POSSUM (risk operative morbidity and mortality) | 7 |
| Charlson comorbidity index (CCI) | 4 |
| NZ risk score | 2 |
| ASOS (Surgical risk score) | 2 |
| Clavien-Dindo | 1 |
| New Injury Severity Score (NISS) | 1 |
| SURPAS (general) | 1 |
| RoS (risk of surgery) | 1 |
|  |  |
| Nutrition |  |
| Nutrition score | 11 |
| SNAQ (nutritional status) | 1 |
| MUST (malnutrition Universal screening tool | 1 |
| HSBLED (risk of bleeding with anticoagulant use) | 1 |
|  |  |
| Thrombosis/bleeding |  |
| Caprini (venous thromboembolism) | 5 |
| Risk of thrombosis (not specified) | 1 |
| Blood transfusion score | 1 |
|  |  |
| Intoxication |  |
| NSiQ (smoking) | 1 |
| Cage (alcohol use) | 1 |
|  |  |
| Geriatric / mental status |  |
| Geriatric screening (unspecified) | 2 |
| G8 (geriatric screening tool) | 2 |
| MOCA score (cognitive assessment) | 1 |
| Cognitive score (not specified) | 1 |
| PMH (positive mental health) | 1 |
| Delirium score | 1 |
|  |  |
| Other |  |
| Apfel score (postoperative nausea and vomiting) | 16 |
| DASI score (activity status) | 7 |
| Metabolic equivalent to ASA | 4 |
| SOFA (sequential organ failure assessment) | 3 |
| POMA (Performance-Oriented Mobility Assessment) | 1 |
| Aspiration risk scoring | 1 |
| ALBI (albumin-bilirubin) score | 1 |
| Hand grip score | 1 |
| Nottingham Score | 1 |
| Prepare score | 1 |
| Ramsay (sedation score) | 1 |
| ACC score | 1 |
| Mil score | 1 |
|  |  |
| **2. Additional clinical assessment tools** |  |
| Functional tests (ECG, spirometry, echo, cardiopulmonary exercise test) | 31 |
| Medical history examination / anamnesis | 24 |
| General assessment by specialist (assessment not specified) | 19 |
| Laboratory tests | 17 |
| Physical examination | 15 |
| Subspecialty evaluation (e.g. cardiologist) | 10 |
| Age | 3 |
| BMI | 3 |
| Airway evaluation | 3 |
| Imaging | 2 |
| Specific biological sample | 2 |
| Multidisciplinary meeting | 2 |
| Patients’ needs are defined for each case separately | 1 |
|  |  |
| **4. Operative / anesthesiological characteristics** |  |
| Type of surgery | 4 |
| Previous anesthesia experience | 3 |
| Regional anesthesia (e.g. inclusion of arterial line) | 1 |
|  |  |
| **5. Other / Unknown** |  |
| Autonomie (France) | 1 |
| Chirurgi carcinologique | 1 |
| MEK | 1 |
| De Gabinete | 1 |
| BW | 1 |
| Vasquip | 1 |
| Only protocols available for joint surgeries | 1 |
| NSO patient risk calculator | 1 |

Respondents who reported using the ASA score, plus additional assessment tools, were asked to write in the text box what additional assessment tools were used (this was not a mandatory question). *One respondent could name multiple items.

**Supplementary Material 4**. Pre-operative assessment tool(s) when the ASA score is not used

|  |  |
| --- | --- |
| \| **Additional tool(s) used** \| **Nr. of respondents reported** \| \| --- \| --- \| \| Medical history (incl. medications, allergies etc.) \| 11 \| \| Medical history and laboratory investigation \| 3 \| \| Medical history, physical examination, laboratory investigation, functional tests (ECG, echo etc.), and other scores \| 3 \| \| General screening by nurse/anesthesiologist (not further specified) \| 3 \| \| Medical history (incl. medications, allergies etc.), airway examination, previous anesthesia complications \| 3 \| \| Medical history (incl. medications, allergies etc.) and previous anesthesia complications \| 2 \| \| Airway score (i.e., Mallampati / total airway score) \| 2 \| \| Medical history and other scores \| 2 \| \| Medical history and physical examination \| 1 \| \| Screening by anesthesiologist only when patient is "flagged" (complex patients) \| 1 \| \| Laboratory tests \| 1 \| \| Too much to document \| 1 \| \| Not reported/answered \| 24 \| |  |
| Respondents that reported not to use the ASA score, were asked to write in the text box what additional assessment tools  were used (this was not a mandatory question). |  |

**Supplementary Material 5**. Beyond top 10 suggestions to add to the ASA classification

| **Suggestions to add to the ASA classification** | **Nr. of times reported*** |
| --- | --- |
| Nutritional status | 33 |
| Clinical assessment | 32 |
| Acute status modifier separate from "Emergent" | 27 |
| More objective criteria | 24 |
| Cardiac risk | 22 |
| Airway assessment | 22 |
| Education/training | 19 |
| Stable or unstable character of diseases | 18 |
| Clarity about malignancy | 17 |
| A scoring system | 17 |
| Need for a different system | 15 |
| Prognosis | 15 |
| Cardiopulmonal status | 14 |
| Experience of treating clinician | 12 |
| A specific pregnancy modifier | 9 |
| Laboratory values | 8 |
| Consideration of the reason for surgery | 8 |
| AI algorithm | 7 |
| Assessment by anesthesiologist | 7 |
| Official (online) calculator | 7 |
| Medication (including previous chemotherapy) | 7 |
| Mental/cognitive assessment | 7 |
| Smoking | 6 |
| Dialysis | 5 |
| More interaction between anesthesia and surgery | 5 |
| Exercise tolerance | 4 |
| Complexity of anesthesiologic plan | 4 |
| Standardization | 4 |
| Do not consider the reason for surgery | 3 |
| Simplification of the score | 3 |
| Changing the remuneration system | 3 |
| Allergies | 2 |
| Doppler evaluation of carotideal stenosis for positioning in MILS | 2 |
| Patient compliance | 2 |
| Two independent assessors | 2 |
| Basic level of debilitation | 1 |
| Cognitive status | 1 |
| Cost of treatment | 1 |
| Frequency of encounters patients have had with healthcare | 1 |
| Frequency of hospitalization | 1 |
| Gender and previous surgeries | 1 |
| Hospital resources | 1 |
| Metabolic equivalents | 1 |
| Mobility at baseline | 1 |
| Need for lines | 1 |
| Predictable difficulties in patient management | 1 |
| Prior anesthetic complications | 1 |
| Pulmonary status | 1 |
| Remove constant threat to life | 1 |
| Sarcopenia | 1 |
| Up to date health record | 1 |
| Acute blood loss | 1 |
| Nothing | 104 |
| Not reported/answered | 123 |

Respondents were asked: what would be important to add to the ASA classification to reduce variability and better reflect true peri-operative risk? *One respondent could name multiple items.

**Supplementary Appendixes**

X

**Supplementary Figures and Tables**

Proceeds on the next page

| **Operation** | **Case** | **ASA score** | **Africa  (n=11)** | **Asia-pacific (n=150)** | **Europe (n=423)** | **North America (n=601)** | **South America (n=96)** | **ALD (%)** | **P value** |
| --- | --- | --- | --- | --- | --- | --- | --- | --- | --- |
| Pancreatoduodenectomy | 1 | ASA 1 | 0 (0%) | 36 (16%) | 5 (9%) | 5 (1%) | 6 (6%) | **24%** | **<0.001** |
|  |  | ASA 2 | 5 (45%) | **293 (65%)** | **280 (69%)** | 280 (47%) | **53 (55%)** |  |  |
|  |  | ASA 3 | **6 (55%)** | 90 (15%) | 295 (21%) | **295 (49%)** | 31 (32%) |  |  |
|  |  | ASA 4 | 0 (0%) | 3 (4%) | 21( 1%) | 21 (3%) | 5 (5%) |  |  |
|  |  | ASA 5 | 0 (0%) | 1 (0%) | 0 (0%) | 0 (0%) | 1 (1%) |  |  |
|  | 2 | ASA 1 | 0 (0%) | 3 (2%) | 0 (0%) | 0 (0%) | 0 (0%) | **38%** | **<0.001** |
|  |  | ASA 2 | 0 (0%) | 59 (39%) | 126 (30%) | 67 (11%) | 30 (31%) |  |  |
|  |  | ASA 3 | **10 (91%)** | **80 (53%)** | **279 (66%)** | **469 (78%)** | **53 (55%)** |  |  |
|  |  | ASA 4 | 1 (9%) | 6 (4%) | 17 (4%) | 63 (10%) | 12 (13%) |  |  |
|  |  | ASA 5 | 0 (0%) | 2 (1%) | 1 (0%) | 2 (0%) | 1 (1%) |  |  |
|  | 3 | ASA 1 | 0 (0%) | 0 (0%) | 1 (0%) | 0 (0%) | 0 (0%) | **12%** | **<0.001** |
|  |  | ASA 2 | 0 (0%) | 14 (9%) | 11 (3%) | 6 (1%) | 1 (1%) |  |  |
|  |  | ASA 3 | **7 (64%)** | **92 (61%)** | **277 (65%)** | **375 (62%)** | **51 (53%)** |  |  |
|  |  | ASA 4 | 2 (18%) | 41 (27%) | 131 (31%) | 220 (37%) | 40 (42%) |  |  |
|  |  | ASA 5 | 2 (18%) | 3 (2%) | 3 (1%) | 0 (0%) | 4 (4%) |  |  |
|  | 4 | ASA 1 | 0 (0%) | 12 (8%) | 22 (5%) | 4 (1%) | 1 (1%) | **30%** | **<0.001** |
|  |  | ASA 2 | **7 (64%)** | **98 (65%)** | **227 (54%)** | 209 (35%) | **47 (49%)** |  |  |
|  |  | ASA 3 | 3 (27%) | 28 (19%) | 151 (36%) | **342 (57%)** | 36 (38%) |  |  |
|  |  | ASA 4 | 1 (9%) | 11 (7%) | 20 (5%) | 43 (7%) | 10 (10%) |  |  |
|  |  | ASA 5 | 0 (0%) | 1 (1%) | 3 (1%) | 3 (0%) | 2 (2%) |  |  |
| Hemihepatectomy | 5 | ASA 1 | 0 (0%) | 11 (7%) | 31 (7%) | 3 (0%) | 1 (1%) | **36%** | **<0.001** |
|  |  | ASA 2 | 3 (27%) | **82 (55%)** | **229 (54%)** | 176 (29%) | **54 (56%)** |  |  |
|  |  | ASA 3 | **7 (64%)** | 50 (33%) | 151 (36%) | **365 (61%)** | 27 (28%) |  |  |
|  |  | ASA 4 | 1 (9%) | 6 (4%) | 12 (3%) | 55 (9%) | 13 (14%) |  |  |
|  |  | ASA 5 | 0 (0%) | 1 (1%) | 0 (0%) | 2 (0%) | 1 (1%0 |  |  |
|  | 6 | ASA 1 | 0 (0%) | 0 (0%) | 3 (1%) | 0 (0%) | 0 (0%) | **26%** | **<0.001** |
|  |  | ASA 2 | 3 (27%) | 69 (46%) | 146 (35%) | 79 (13%) | 36 (38%) |  |  |
|  |  | ASA 3 | **7 (64%)** | **71 (47%)** | **245 (58%)** | **441 (73%)** | **45 (47%)** |  |  |
|  |  | ASA 4 | 1 (9%) | 8 (5%) | 29 (7%) | 81 (13%) | 14 (15%) |  |  |
|  |  | ASA 5 | 0 (0%) | 2 (1%) | 0 (0%) | 0 (0%) | 1 (1%) |  |  |
|  | 7 | ASA 1 | 0 (0%) | 2 (1%) | 7 (2%) | 0 (0%) | 0 (0%) | **34%** | **<0.001** |
|  |  | ASA 2 | 3 (27%) | **78 (52%)** | 170 (40%) | 95 (16%) | 38 (40%) |  |  |
|  |  | ASA 3 | **8 (73%)** | 59 (39%) | **227 (54%)** | **432 (72%)** | **46 (48%)** |  |  |
|  |  | ASA 4 | 0 (0%) | 9 (6%) | 17 (4%) | 74 (12%) | 11 (11%) |  |  |
|  |  | ASA 5 | 0 (0%) | 2 (1%) | 2 (0%) | 0 (0%) | 1 (1%) |  |  |
|  | 8 | ASA 1 | 1 (9%) | 8 (5%) | 32 (8%) | 5 (1%) | 2 (2%) | **31%** | **<0.001** |
|  |  | ASA 2 | 4 (36%) | **84 (56%)** | **236 (56%)** | 187 (31%) | **53 (55%)** |  |  |
|  |  | ASA 3 | **5 (45%)** | 50 (33%) | 142 (34%) | **367 (61%)** | 29 (30%) |  |  |
|  |  | ASA 4 | 1 (9%) | 6 (4%) | 12 (3%) | 42 (7%) | 11 (11%) |  |  |
|  |  | ASA 5 | 0 (0%) | 2 (1%) | 1 (0%) | 0 (0%) | 1 (1%) |  |  |

**Supplementary material 6.** Differences in ASA scores among regions

Bold numbers indicate the highest scores among regions, and indicate clinical relevance or statistical significance.

**Supplementary material 7.** Interrater variability within continents

| **Case** | **Africa  (n=11)** | **Asia-pacific (n=150)** | **Europe (n=423)** | **North America (n=601)** | **South America (n=96)** |
| --- | --- | --- | --- | --- | --- |
| 1 | No agreement K = -0.091 | Fair agreement K = 0.287 | Moderate agreement K = 0.414 | Fair agreement K = 0.278 | Fair agreement K = 0.262 |
| 2 | Substantial agreement K = 0.636 | Fair agreement K = 0.297 | Fair agreement 49% K = 0.366 | Moderate agreement K = 0.509 | Fair agreement K = 0.262 |
| 3 | Slight agreement K = 0.127 | Fair agreement K = 0.275 | Fair agreement K = 0.405 | Fair agreement K = 0.284 | Fair agreement  K = 0.269 |
| 4 | Slight agreement K = 0.155 | Fair agreement K = 0.337 | Fair agreement K = 0.274 | Fair agreement K = 0.311 | Fair agreement K = 0.232 |
| 5 | Slight agreement K = 0.155 | Fair agreement K = 0.266 | Fair agreement K = 0.234 | Fair agreement K = 0.266 | Fair agreement K = 0.266 |
| 6 | Slight agreement K = 0.155 | Fair agreement K = 0.247 | Fair agreement K = 0.277 | Fair agreement K = 0.360 | Slight agreement K = 0.167 |
| 7 | Slight agreement K = 0.127 | Fair agreement K = 0.282 | Fair agreement K = 0.313 | Fair agreement K = 0.334 | Slight agreement K = 0.191 |
| 8 | No agreement K = 0.055 | Fair agreement K = 0.282 | Fair agreement  K = 0.286 | Fair agreement K = 0.298 | Fair agreement  K = 0.254 |

**Supplementary material 8.** Difference in ASA score among surgeons and anesthesiologists

| **Operation** | **Case** | **ASA score** | **Anesthesiologist** | **Surgeon** | **ALD** | **P value** |
| --- | --- | --- | --- | --- | --- | --- |
| Pancreatoduodenectomy | 1 | ASA 1 | 30 (2%) | 41 (20%) | 6% | **<0.001** |
|  |  | ASA 2 | **600 (56%)** | **130 (62%)** |  |  |
|  |  | ASA 3 | 409 (38%) | 36 (17%) |  |  |
|  |  | ASA 4 | 33 (3%) | 2 (1%) |  |  |
|  |  | ASA 5 | 1 (0%) | 1 (0%) |  |  |
|  | 2 | ASA 1 | 1 (0%) | 2 (0%) | **23%** | **<0.001** |
|  |  | ASA 2 | 192 (18%) | 91 (43%) |  |  |
|  |  | ASA 3 | **786 (73%)** | **106 (50%)** |  |  |
|  |  | ASA 4 | 89 (8%) | 10 (5%) |  |  |
|  |  | ASA 5 | 5 (0%) | 1 (5%) |  |  |
|  | 3 | ASA 1 | 1 (0%) | 0 (0%) | 5% | 0.069 |
|  |  | ASA 2 | 21 (2%) | 11 (5%) |  |  |
|  |  | ASA 3 | **680 (64%)** | **123 (59%)** |  |  |
|  |  | ASA 4 | 361 (34%) | 74 (35%) |  |  |
|  |  | ASA 5 | 10 (1%) | 2 (1%) |  |  |
|  | 4 | ASA 1 | 15 (1%) | 24 (11%) | **20%** | **<0.001** |
|  |  | ASA 2 | 475 (44%) | **114 (54%)** |  |  |
|  |  | ASA 3 | **505 (47%)** | 56 (27%) |  |  |
|  |  | ASA 4 | 71 (7%) | 14 (7%) |  |  |
|  |  | ASA 5 | 7 (1%) | 2 (1%) |  |  |
| Hemihepatectomy | 5 | ASA 1 | 14 (1%) | 33 (16%) | **26%** | **<0.001** |
|  |  | ASA 2 | 428 (40%) | **116 (55%)** |  |  |
|  |  | ASA 3 | **549 (51%)** | 52 (25%) |  |  |
|  |  | ASA 4 | 78 (7%) | 9 (4%) |  |  |
|  |  | ASA 5 | 4 (0%) | 0 (0%) |  |  |
|  | 6 | ASA 1 | 0 (0%) | 3 (1%) | **30%** | **<0.001** |
|  |  | ASA 2 | 3 (21%) | **109 (52%)** |  |  |
|  |  | ASA 3 | **7 (68%)** | 80 (38%) |  |  |
|  |  | ASA 4 | 1 (11%) | 18 (9%) |  |  |
|  |  | ASA 5 | 0 (0%) | 0 (0%) |  |  |
|  | 7 | ASA 1 | 2 (0%) | 7 (3%) | **28%** | **<0.001** |
|  |  | ASA 2 | 273 (25%) | **112 (53%)** |  |  |
|  |  | ASA 3 | **695 (65%)** | 78 (37%) |  |  |
|  |  | ASA 4 | 100 (9%) | 11 (5%) |  |  |
|  |  | ASA 5 | 3 (0%) | 2 (0%) |  |  |
|  | 8 | ASA 1 | 20 (2%) | 28 (13%) | **27%** | **<0.001** |
|  |  | ASA 2 | 443 (41%) | **122 (58%)** |  |  |
|  |  | ASA 3 | **543 (51%)** | 51 (24%) |  |  |
|  |  | ASA 4 | 63 (6%) | 9 (4%) |  |  |
|  |  | ASA 5 | 4 (0%) | 0 (0%) |  |  |

Bold numbers indicate the highest scores among anesthesiologists and surgeons, and indicate clinical relevance or statistical significance.

**References**

No additional references
